# cAMP Compartmentalization in Cerebrovascular Endothelial Cells: New Therapeutic Opportunities in Alzheimer’s Disease

**DOI:** 10.3390/cells10081951

**Published:** 2021-07-31

**Authors:** Dolores Viña, Nuria Seoane, Elisardo C. Vasquez, Manuel Campos-Toimil

**Affiliations:** 1 Physiology and Pharmacology of Chronic Diseases (FIFAEC), Molecular Medicine and Chronic Diseases Research Centre (CIMUS), University of Santiago de Compostela, 15782 Santiago de Compostela, Spain; mdolores.vina@usc.es (D.V.); nuria.seoane@rai.usc.es (N.S.); 2 Pharmaceutical Sciences Graduate Program, Vila Velha University, Vila Velha 29102-920, ES, Brazil; evasquez@terra.com.br

**Keywords:** adenylyl cyclases, AKAP, Alzheimer’s disease, blood brain barrier, endothelium, cyclic AMP, Epac proteins, intracellular compartmentalization, phosphodiesterase, protein kinase A

## Abstract

The vascular hypothesis used to explain the pathophysiology of Alzheimer’s disease (AD) suggests that a dysfunction of the cerebral microvasculature could be the beginning of alterations that ultimately leads to neuronal damage, and an abnormal increase of the blood–brain barrier (BBB) permeability plays a prominent role in this process. It is generally accepted that, in physiological conditions, cyclic AMP (cAMP) plays a key role in maintaining BBB permeability by regulating the formation of tight junctions between endothelial cells of the brain microvasculature. It is also known that intracellular cAMP signaling is highly compartmentalized into small nanodomains and localized cAMP changes are sufficient at modifying the permeability of the endothelial barrier. This spatial and temporal distribution is maintained by the enzymes involved in cAMP synthesis and degradation, by the location of its effectors, and by the existence of anchor proteins, as well as by buffers or different cytoplasm viscosities and intracellular structures limiting its diffusion. This review compiles current knowledge on the influence of cAMP compartmentalization on the endothelial barrier and, more specifically, on the BBB, laying the foundation for a new therapeutic approach in the treatment of AD.

## 1. Introduction

Median age increases, especially in developed countries, are accompanied by an increase in the prevalence of several types of primary degenerative dementias, most of them associated with the elderly. However, differential diagnoses are difficult, since their etiopathogenic characteristics, as well as clinical behaviors, often overlap and become confusing.

Cerebrospinal fluid (CSF) is a clear, plasma-like fluid (an ultrafiltrate of plasma) that surrounds the brain and spinal cord. Recent studies have demonstrated the usefulness of CSF biomarkers in distinguishing between dementias in routine clinical settings, as well as indicating the presence of AD. Markers to consider in CSF are Aβ42 (sometimes normalized to the related peptide Aβ40), total tau (t-tau) protein, and phosphorylated tau (p-tau) protein. They make it possible to distinguish between AD, frontotemporal dementia, Lewy body dementia, Parkinson’s disease dementia, vascular dementia, and mixed dementia (AD and vascular) [1].

Currently, it is possible to establish that AD is the most common cause of dementia, accounting for an estimated 60% to 80% of cases [2], and the absence of an effective treatment, as well as the poor success in recent drug development efforts, is a growing health and social problem. For this reason, in recent years, intensive research has been carried out to understand the molecular mechanisms of AD and, based on this, to develop possible treatments.

AD pathogenesis is attributed to the loss of neuronal cells and the progressive atrophy of nervous tissue. However, neuronal loss shows important distinctions between different cell populations, the most affected being cholinergic neurons, which use acetylcholine (ACh) as a neurotransmitter. ACh is involved in various, higher cognitive processes, such as attention, learning, and memory. Moreover, cholinesterase inhibitors, such as donepezil, galantamine, and rivastigmine, have been commonly prescribed for AD for decades. However, although the inhibition of this esterase shows certain benefits, these drugs only provide temporary or incomplete symptomatic relief, and are not able to effectively slow the progression of AD [3].

In the hippocampus, neuronal dysfunction can be derived from excitotoxicity, caused by consistently elevated glutamate levels, which cause excessive activation of the *N*-methyl-d-aspartate (NMDA) glutamate receptor, or from an increased sensitivity to glutamate, resulting in enhanced Ca^2+^ flux reaching neurons, impaired neuronal homeostasis, and neurodegeneration [4]. This justifies the use of memantine, a NMDA receptor antagonist for AD treatment.

Various histopathological and functional alterations that occur during AD progression are widely known. The most prominent include the formation of amyloid-β (Aβ) deposits and neurofibrillary tangles of p-tau protein in the neuronal framework, both used as markers in AD diagnosis, or the involvement of inflammatory processes.

The Aβ peptide comes from the aberrant processing of a neuronal transmembrane protein, known as amyloid precursor protein (APP). The key step in this pathway is the cleavage by β-secretase at the *N*-terminus of Aβ, followed by another cleavage catalyzed by γ-secretase, resulting in the formation of Aβ oligomers (Aβ40 and Aβ42) that will polymerize, the neurotoxicity caused by Aβ42 being much greater [5]. This aggregation entails, as a result, the blocking of ionic channels, the alteration of calcium homeostasis, an increase in mitochondrial oxidative stress, and a decrease in energy metabolism and glucose regulation, which contributes to the deterioration of neuronal health and, finally, to the death of neuronal cells [6].

Another pathological feature of AD consists of the neurofibrillary changes represented by neurofibrillary tangles and neurofibrillary threads, which exhibit a stereotyped pattern of hierarchical progression initiated around the hippocampus. The hyperphosphorylation of tau, whose oligomers aggregate to form the neurofibrillary tangles, causes instability and collapse of the microtubules, loss of communication between neurons and, finally, neuronal apoptosis [6]. Recent research data supports the theory that correlates the extent of neurofibrillary changes to the severity of dementia in AD [7].

Therefore, aberrant proteins (abovementioned) are clearly associated with altered neurotransmission, neurodegeneration processes, and atrophy of brain structures. However, there are still numerous concerns about the causal sequence and interrelation of these events.

Considering the importance of the blood–brain barrier (BBB) to protect the central nervous system (CNS), the vascular hypothesis of the origin of AD has gained strength in the last two decades. It complements other existing theories, proposing that initial vascular damage precipitates AD. However, although the mechanisms linking BBB disruption and neurodegeneration provide an interesting basis for the search for new therapies for neurodegenerative diseases [8], the precise mechanisms of BBB impairment are not fully understood.

It is generally accepted that 3′,5′-cyclic adenosine monophosphate (cAMP) signaling participates in a prominent way in the regulation of endothelial permeability. An increase in cAMP concentrations improves the functionality of the endothelial barrier and increases tight junction (TJ) resistance in BBB [9,10]. Therefore, better knowledge of the local processes regulated by cAMP may be key when it comes to preserving the BBB function and preventing the progression of neurodegenerative diseases through pharmacological intervention.

This review proposes, for the first time, the possibility of modulating cAMP signaling pathways to preserve the cerebrovascular endothelial barrier function as a new strategy to prevent BBB hyperpermeability in neurodegenerative dementia, according to the vascular theory of AD. Our aim herein is to update knowledge on the role of cAMP and its complex and highly compartmentalized signaling in BBB disruption and propose new therapeutic targets for AD treatment.

## 2. The Vascular Hypothesis of Alzheimer’s Disease (AD)

In the search for new hypotheses that seek to explain the origins of neurodegenerative dementias, we considered that, in the elderly, vascular disorders make the brain more vulnerable, intensifying the symptoms of neurodegenerative disease.

From the observation that AD patients present reduced cerebral blood flow, cerebral oxygen consumption, and glucose metabolism, proportional to the severity of the disease, the vascular hypothesis has emerged as an explanation for the origin of AD [11]. In accordance with this hypothesis, vascular and circulatory alterations in the brain precede the appearance of clinical symptoms by years or decades [12]. In fact, post-mortem studies suggest that more than half of AD patients had cerebrovascular abnormalities [13]. Moreover, clinical, imaging, neuropathological, and epidemiological evidence have been accumulated, confirming a key role of cerebrovascular disease in AD and other age-associated dementias [14].

Currently, vascular dysfunction is considered a key factor in the development of AD. However, it is not yet clear whether vascular abnormalities precede or follow AD pathology, and the role of microcirculation in neurodegenerative disorders has not been sufficiently considered until now. This has probably contributed significantly to the failure of clinical trials, with various neuroprotective drugs.

A systematic review of the literature showed that a variety of disorders affecting small arteries and microvessels of the brain are associated with AD and may be related to Aβ/tau pathology [15]. Thus, a large body of evidence has been presented to show that vascular disorders are involved in the pathogenesis of AD. A study demonstrated the prevalence of large infarcts, multiple lacunae and microinfarctions, hemorrhages, atherosclerosis, and arteriolosclerosis in 80% of patients diagnosed with AD [16]. In addition, several studies have shown that vascular risk factors, such as dyslipidemia and hypertension, contribute to the development of AD [17]. Although there have been few advances (in terms of therapy) in this regard, long-term treatment with the anticoagulant dabigatran improves cerebral perfusion and BBB function, preserving cognitive function and demonstrating the potential of a cardiovascular treatment to delay AD progression [18].

Even in patients suffering from the genetic form of AD, many functional studies support the vascular hypothesis that drives the AD phenotype. A degenerative response of endothelial cells and pericytes mediated by cyclophilin A and nuclear factor kappa B (NF-κB) were observed in mice expressing human APOE4, the main genetic risk factor for AD [19]. This response is also seen in APOE4-bearing humans. Another fact that supports the idea that the first changes in the BBB could play a main role in the initiation of AD is that the heterozygous deficiency of glucose transporter 1 (GLUT1) accelerates the degenerative changes in the endothelium in the APPsw mouse model for AD [20].

Vascular dysfunction has a complex etiology (e.g., it is related to an increase in oxidative stress and inflammation). Mediators that are released have negative effects on the brain’s microvascular endothelial cells forming the BBB, as well as on other components of the neurovascular unit (NVU), such as astrocytes or microglia, causing an abnormal increase in permeability [8]. Vascular damage also influences the Aβ-mediated neurodegeneration pattern, as the resulting BBB dysfunction leads to poor clearance of this peptide, contributing to its accumulation in the brain and the subsequent neurotoxic effects [21].

The integrity of the NVU is necessary for the proper functioning of the brain, and damage at this level can lead to different diseases that affect the CNS. Currently, there is extensive evidence that NVU dysfunction contributes to AD [22].

## 3. Disruption of Blood–Brain Barrier (BBB) in AD

The vascular hypothesis of AD suggest that the BBB plays a key role in the pathogenesis of Alzheimer’s disease. The BBB is a cell membrane that covers brain microvessels. It is composed of vascular endothelial cells of the microvasculature of the CNS, which communicate with other cells of the CNS, such as astrocytes, pericytes, and neurons, constituting the NVU [23,24].

Regarding the whole vascular system, the endothelium constitutes the innermost layer of all blood vessels, and one of its most prominent functions is the formation of a structural barrier that, through TJs, regulates the exchange of solutes between tissues and the blood, and the extravasation of multiple substances, platelets, and blood cells [25,26]. It also plays a crucial role in the control of multiple important functions, acting as an endocrine organ. Among its functions is the regulation of the regional blood flow, controlling vascular tone by releasing various vasodilator molecules, such as nitric oxide (NO), prostacyclin (PGI2), or the endothelium-derived hyperpolarizing factor (EDHF), and contracting factors, such as endothelins [27,28]. It also participates in the regulation of inflammation and angiogenesis [29]. Therefore, it is not surprising that endothelial alterations are implicated in the pathogenesis and evolution of a wide spectrum of diseases [25,30,31,32].

Each NVU component plays an important role in maintaining the proper functioning of the BBB. Endothelial cells express multiple substrate-specific transport systems, which control the passage of essential molecules from the blood to the brain, as well as the transport of metabolic waste products from the interstitial fluid to the blood. In addition, endothelial cells are connected to each other by TJs, which link contiguous cells by multiple transmembrane proteins. This compact structure endows the endothelium with low permeability, which is reinforced by a covering formed by pericytes and astrocyte feet. Moreover, the pre-conditioning of human umbilical vein endothelial cells (HUVEC) with astrocyte-conditioned medium reduces the permeability to levels similar to that of the BBB [33]. Microglia cells act as the first line of defense, while pericytes act by regulating angiogenesis, toxin elimination, brain flow, and entry of immune cells through the BBB. For their part, astrocytes guarantee brain metabolism, regulate synaptic transmission and plasticity, and prevent neuronal toxicity [34,35,36].

The BBB allows the selective passage of nutrients and energy to the brain, which are essential for neuronal function and preventing the entry of neurotoxic substances from the peripheral circulation. Therefore, BBB has a crucial role in the maintenance of homeostasis in the CNS by limiting transport of toxic or harmful molecules, transport of nutrients, and removal of metabolites from the brain [24,28]. Transport through the endothelial cells is mainly mediated by the expression of receptors and transporters, such as the receptor for advanced glycosylation end products (RAGE), low-density lipoprotein receptor-related protein 1 (LRP-1), and P-glycoprotein (P-gp) [37].

In certain pathological situations, the endothelium may undergo dysfunction, and its permeability may increase abnormally [38]. Consequently, an endothelial dysfunction at the cerebral microvasculature may lead to a pathological fenestration of the BBB, with the subsequent abnormal increase of the barrier permeability and neuronal damage [39].

It is generally accepted that BBB dysfunction occurs in AD, even before neurodegeneration and dementia. In fact, an increase in the normally low permeability of the BBB appears in the pathogenesis of various neurological diseases, and is present before the appearance of the first clinical symptoms [23,24].

The brains of AD patients were found to have lower expressions of various proteins involved in the formation of the TJs of the BBB endothelial cells, such as claudin-5, occludin, and zonula occludens-1 (ZO-1) [40,41]. Moreover, an increase in endothelial RAGE reactivity has been found in these patients, while the expression of LRP-1 and the function and expression of P-gp are reduced, decreasing the clearance of Aβ peptide from the brain [37].

BBB disruption leads to the entry of blood constituents into the CNS, alters the clearance mechanism, and is associated with reduced cerebral flow. Recently, it was also shown that, at the endothelial level, cerebral blood flow can be regulated by GLUT1. GLUT1 is further required to maintain the integrity of BBB and proper brain capillary networks, as well as neuronal function. Its deficiency in endothelium initiates the vascular phenotype of AD, as shown by BBB breakdown. In GLUT1-deficient AD mice (Slc2a1+/− APPSw/0), reduced brain perfusion and diminished glucose uptake into the brain occur at 2 weeks of age. However, neuronal dysfunction, behavioral deficits, elevated Aβ levels, and behavioral and neurodegenerative changes take approximately 6 months to develop [20]. Based on these (and other) results, BBB disruption is currently considered an early indicator of cognitive dysfunction and AD.

**Figure 1:**
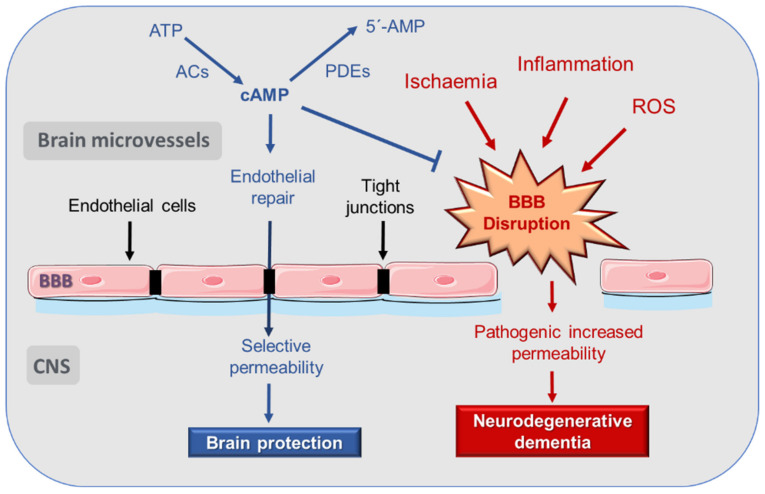
cAMP in the vascular theory of Alzheimer disease. cAMP exerts a protective effect on the integrity of the endothelium in the blood–brain barrier against processes that cause its pathological fenestration and the development of neurodegenerative diseases. ACs: adenylyl cyclases; BBB: blood brain barrier; CNS: central nervous system; PDE: phosphodiesterase; ROS: reactive oxygen species. For more details, see text.

Consequently, the mechanisms that relate BBB disruption and neurodegeneration constitute an interesting basis in the search for new therapies for neurodegenerative diseases (Figure 1).

Thus far, the precise mechanisms responsible for BBB impairment are not fully understood. Research has focused on the role played by both the pericytes and the endothelial cells of the brain microvasculature in BBB disruption. In post-mortem studies, it was observed that a degeneration of the pericytes occurs in regions that presented elevated fibrillar Aβ, which induces or worsens BBB disruption [42]. Likewise, it has been experimentally shown that apolipoprotein E (APOE), a risk factor for AD, activates the pro-inflammatory cyclophilin A-nuclear factor-κM-matrix-metalloproteinase-9 pathway in pericytes, leading to BBB impairment [19].

Regarding endothelial dysfunction, aging is an independent factor, even in the absence of other cardiovascular risk factors. Vascular inflammation occurs with aging [43,44] and can be reinforced by metabolic and cardiovascular diseases [45,46]. Thrombin is an important mediator that produces vascular inflammation. It can directly activate endothelial cells and promote the expression of pro-inflammatory proteins, such as intercellular adhesion molecule-1 (ICAM-1) and monocyte chemoattractant protein-1 (MCP-1), the release of angiopoietin-2, and the positive regulation of αVβ3 integrin [47]. Inflammatory mediators interact with leukocytes and reduce vasodilation-modifying BBB permeability [48].

There are several sources of reactive oxygen species (ROS), which are altered in AD, such as mitochondrial electron-transport chain, cyclooxygenases (COXs), lipoxygenases, cytochrome P450 reductases, xanthine oxidase, nitric oxide synthase (NOS), and Nox. ROS produce oxidative stress and are responsible for altering protein structure, DNA denaturation, and lipid peroxidation, and may act as messengers in redox-signaling systems. Superoxide, hydroxyl radical, and hydrogen peroxide are common ROS with deleterious effects on the vascular endothelium. Their concentrations depend on the balance between oxidases, such as nicotinamide adenine dinucleotide phosphate (NADPH) oxidases (Nox enzymes) and superoxide dismutase (SOD). The impact of ROS on BBB function was demonstrated in SOD deficient mice, in which ischemia/reperfusion experiments demonstrated an enhanced endothelial permeability to large molecules [49].

Claudin-5 expression is regulated by ROS production, increasing the leakage of solute, and modifying the BBB integrity [50]. Similarly, occludin expression is reduced by AMP-activated protein kinase (AMPK) activation that enhances lipopolysaccharide (LPS)-impaired BBB functions through suppression of NADPH oxidase-derived ROS in mice [51]. In addition to claudin and occludin, ROS can change ZO protein distribution. Exposure to hydrogen peroxide has been reported to lead to the redistribution of ZO-1 from the TJs to the cytosol, resulting in decreased transepithelial electric resistance (TEER) and increased BBB permeability [52]. Therefore, ROS cause changes in various parameters that compromise the integrity of the BBB.

The BBB damage produced by ROS is also related to the activation of transcription factors that produce a proinflammatory response, with NF-κB being the main regulator. Activation of NF-κB by ROS can increase ICAM-1 and vascular cell adhesion molecule 1 (VCAM-1) expression [53]. ICAM-1 can activate a Ca^2+^ signaling pathway that can lead to cytoskeleton changes in microvascular endothelial cells of the brain, causing BBB damage [54].

ROS overproduction also interferes with hypoxia-inducible factor 1α (HIF-1α) reducing its expression and activity. Thus, the stimulus to promote angiogenesis and the formation of new vessels will be restricted, leading to a vicious cycle of impaired capillary perfusion, hypoxia, and oxidative stress [55].

It follows—from the above—that a highly selective BBB permeability is essential for the maintenance of healthy brain functioning. In this sense, it is known that the cAMP signaling pathway plays a very prominent role in the regulation of cerebrovascular endothelial permeability, as reviewed in the following sections.

## 4. cAMP Regulation of Endothelial and BBB Permeability

cAMP is a second universal messenger, generated when an extracellular first messenger (neurotransmitters, hormones, chemokines, lipid mediators, or drugs) binds to a G-protein coupled receptor (GPCR) associated with an AC enzyme that catalyzes the cyclization of adenosine triphosphate (ATP) to generate cAMP [56,57]. G proteins that regulate the intracellular concentration of cAMP are of two subtypes: Gs or Gi proteins, which stimulate or inhibit, respectively, the activity of adenylyl cyclases (ACs). cAMP intracellular concentration is also regulated by the family of phosphodiesterases (PDEs), which catalyze cAMP hydrolysis, ending the signaling pathway [58].

cAMP participates in the regulation of many biological processes and cellular functions, such as metabolism, gene regulation, regulation of neurotransmitter synthesis, growth factors, and immune function [59].

In the late 1990s, it was suggested that increases in cAMP concentration could be involved in a decrease in endothelial permeability [60] (Figure 1). By measuring microvascular hydraulic conductivity in mesentery of pithed frogs, it could be seen that cAMP decreases microvascular permeability in vivo by increasing the number of Tj strands between endothelial cells [61]. Later studies served to demonstrate that cAMP signaling participates in a prominent way in the regulation of endothelial permeability, and a rise in its concentration enhances barrier functions [10,25,62,63,64,65]. This ability of cAMP to stabilize the barrier occurs in resting conditions and in the presence of barrier destabilizers [66].

In regard to BBB—in 1991, a study using a new in vitro model of BBB, combining brain endothelial cells monolayers and astrocyte conditioned medium, demonstrated that cAMP-elevating agents increase TJ resistance. On the contrary, agents that decreased cAMP concentration or block activity reduced TJ resistance [9]. A few years later, it was shown that various agents that increase cAMP (dibutyryl-cAMP, isoprenaline, and human α-calcitonin gene-related peptide) counteract permeability increases in pial venular capillaries, which are part of the BBB [67].

The in vivo temporal regulation by cAMP of the BBB permeability to solutes was demonstrated in 2014 using multiphoton microscopy in rat brain parenchyma [68]. It was shown that cAMP increase by stimulation of adenosine receptors induces gap junction coupling in human cerebral microvascular endothelial cells, an effect mediated by cyclic nucleotide-gated (CNG) channel activation and the subsequent increase in Ca^2+^ influx [69].

cAMP-activated pathways make up complex and highly compartmentalized signaling. Thus, it is no surprise that study results have also reported that, contrary to the widely accepted notion, an endothelial increase of cAMP could enhance vascular permeability, an effect that would be mediated by transcriptional small guanosine triphosphate hydrolase Ras-related protein (RRAS) suppression [70].

In general, cAMP-mediated signaling can be very different, depending on the cell model. As an example, an increase in cAMP causes contraction of cardiac myocytes, but relaxation of vascular ones. In addition, there are also differences in the cAMP-elicited response depending on the microdomain in which its elevation occurs within the same cell type. Therefore, in the following section, we will address the current knowledge about cAMP compartmentalization, focusing on how it can affect the control of the permeability at the endothelium and the BBB.

## 5. cAMP Compartmentalization

Although initially it was assumed that cAMP was distributed homogeneously in all cell compartments, it is now widely accepted that it is located in independent domains. Thus, cAMP does not activate a linear signaling cascade but, on the contrary, is involved in the activation of a complex network of spatially and temporally regulated signaling pathways in an independent way. In this compartmentalized model, signals with physiological significance occur in confined nanodomains and not through homogeneous changes in the cytosolic cAMP [71,72,73].

The enzymatic components that participate in the generation of cAMP and in the subsequent transduction of its signal have different isoforms, and are controlled by specific regulatory mechanisms. These components contribute to the control of the spatial and temporal compartmentalization of cAMP. Because of this, cAMP-activated signal transduction mechanisms can lead to different responses, depending on the cell area in which they take place [71,72,73,74].

### 5.1. Methods for Studying the cAMP Compartmentalization

The study of compartmentalized cAMP signaling was not possible by using end-point techniques that required cell lysate or by employing cAMP-sensitive molecules whose injections could interfere with normal cell function. Since 1991, using the technique called Föster resonance energy transfer (FRET), it has been possible to create biosensors that allow the study of cAMP signaling in highly localized cellular domains. FRET uses distance-dependent energy transfer from a donor molecule to an acceptor molecule, thereby allowing the determination of the proximity between two molecules at a distance that allows for molecular interactions.

The first cAMP sensor based on the FRET technique was called FlCRhR, and it was based on the labeling of the catalytic and regulatory subunits of protein kinase A (PKA) with two different fluorescent markers, fluorescein and rhodamine [75]. Subsequently, other highly useful sensors were developed for the performance of cAMP signal sublocation experiments, such as the one that uses mutants of the green fluorescent protein (GFP) fused to the different subunits of PKA [76] or those based on the cAMP-binding domains of the exchange proteins directly activated by cAMP (Epac) [77]. More recently, visualization of three-dimensional spatial gradients of cAMP concentration in individual cells was achieved by implementing spectral imaging approaches to estimate FRET efficiency when using multiple fluorescent markers [78,79].

In addition to FRET, other approaches were used in recent years to measure localized variations in cAMP concentrations, including fluorescent proteins that modify their fluorescence intensity based on their binding to cAMP, single luciferase-based cAMP sensors, or bioluminescence resonance energy transfer (BRET)-based cAMP sensors [80]. Moreover, new probes have been designed that can detect variations in the concentration of cAMP at a micro or even nanoscale. The main problem with some of these sensors may be due to a different physiological behaviors or to the different affinities that they may have for cAMP in relation to the unlabeled molecule, which sometimes makes the interpretation of the results difficult. For recent and detailed reviews of cAMP measurement techniques, see, for example, Zaccolo et al. [73], Kim et al. [80], Ghigo and Mika [81], Judina et al. [82], or Chao et al. [83].

These techniques have allowed for better understanding of the spatial regulation of cAMP and the implication in cellular functionality. Thus, they have made it possible to study cAMP compartmentalization in a wide range of cellular models, including cardiomyocytes [81,82], adipocytes [84], vascular smooth muscle cells [85], neurons [86], or astrocytes [87]. However, despite the great utility of these techniques in studying cell signaling compartmentalization, it should be kept in mind that they are techniques that greatly interfere with normal cell functions, so all results should be viewed with caution, and completed, if possible, with parallel functional studies.

In any case, it should be noted that other techniques have also contributed to the study of compartmentalization of intracellular signaling, among which is co-immunoprecipitation [88], a technique that has also been applied to better understand the compartmentalization of cAMP signaling [89,90].

### 5.2. cAMP Signalosomes in Endothelium and BBB

The mechanisms by which cAMP signaling are compartmentalized are not yet fully understood. The general “trend” is to admit that spatial distribution of cAMP signaling is controlled by the enzymes that regulate its levels. In this sense, the grouping of membrane receptors, ACs, and PDEs in lipid rafts, and caveolae, are relevant for the maintenance of spatiotemporal compartmentalized cAMP signaling. Moreover, the localization of the cAMP-activated effectors, such as PKA or Epac, in different domains, contribute to the creation of independent signaling pathways within the same cell—all of this regulated, in turn, by anchor proteins [57,91].

Other aspects that may participate in the complexity of this signaling have been described. It was suggested that the subcellular locations of PDE and AC activities are not sufficient in generating cAMP gradients with physiological relevance and that it is unlikely that compartmentalization depends exclusively on the assembly of these enzymes, and the cAMP effectors onto certain protein scaffolds. The local specificity of this signaling pathway is also based on variations in the effective rate of diffusion of cAMP, which depends on the presence of buffers, localized changes in cytoplasmic viscosity, or the existence of intracellular structural impediments [62,92].

To date, a considerable number of experimental studies and reviews have focused on the description of the different cAMP signalosomes (see, i.e., [73,93,94,95]). Zaccolo et al. [73], in their 2021 review, have comprehensively compiled the existing knowledge in this regard, identifying the different nanodomains of cAMP and their regulations, providing a detailed description of the existing compartments in the plasma membrane, primary cilium, mitochondria, Golgi apparatus, centrosome, endo/sarcoplasmic reticulum, and nucleus.

However, knowledge about how cAMP signalosomes are distributed in the endothelium and, more specifically, in cerebrovascular endothelial cells, is scarce. At present, there are few studies that identify subcellular cAMP domains and their roles in endothelial permeability control. Most of the progress in this regard has been made at the University of South Alabama by Dr. Sarah Sayner, Dr. Troy Stevens, and co-workers, using pulmonary endothelial cells. Based on several previous observations, such as the fact that small, highly localized cAMP changes in microdomains of the plasma membrane were sufficient at increasing the protective properties of the endothelial barrier [96], the importance of the intracellular location of cAMP increases, in determining their effects on endothelial permeability, was first described by Sayner et al. [97]. They showed that the subcellular localization of the cAMP increases was essential in determining its subsequent effects on the endothelial barrier. This study, and subsequent ones, led to an understanding of how the compartmentalized microdomains of cAMP are of critical importance in the functioning of the endothelial barrier function [98,99,100,101].

Thus, this group suggested the existence of two intracellular domains of cAMP in relation to endothelial permeability, and hypothesized about the different roles of plasma membrane cAMP versus cytosolic cAMP in regulating the endothelial barrier. While the former has a protective role, the latter acts primarily as a disruptor [102,103] (see also Section 6.1.1). More recently, a new compartment of cAMP using PMVECs has been described. It is a second extracellular compartment, consisting of encapsulated cAMP within extracellular vesicles, whose function has not yet been determined [104].

Regarding BBB, despite the interest in studying the microdomains of cAMP, and how they participate in the regulation of endothelial function, there are hardly any studies on the intracellular localization of cAMP signaling in cerebrovascular endothelial cells. As described above, the endothelium at the NVU plays a key role in regulating BBB permeability. Therefore, better knowledge of the local processes regulated by cAMP may be key when it comes to preserving the barrier functioning and preventing the progression of neurodegenerative diseases through pharmacological intervention.

In the following section, we review the molecules that regulate the of cAMP compartmentalization, focusing on what happens in the endothelium and the BBB, in order to identify possible drug targets that allow the preservation of the barrier function in the NVU.

## 6. Regulation of the cAMP Compartmentalization at the Endothelium and the BBB

### 6.1. cAMP Generation and Degradation: ACs and PDEs

#### 6.1.1. Adenylyl Cyclases (ACs)

The AC enzyme family is responsible for the synthesis of cAMP production from available ATP. Mammalian ACs are class III enzymes and there are 10 isoforms, of which 9 of them (AC1-9) located in the plasma membrane and only one soluble isoform (AC10 or sAC) is located in the cytoplasm [105]. The structure and function of ACs in mammalian cells have been the subjects of various reviews in recent years [56,106].

Transmembrane ACs are activated by binding of an agonist to a GPCR coupled to a heterotrimeric Gs protein and are inhibited by ligands stimulating GPCR coupled to a Gi protein [107]. Moreover, their activity is regulated by other signaling pathways, including calcium signaling, subunits of other G proteins, and receptor tyrosine kinases.

Concerning endothelial permeability, it has long been known that the activation of Gs-coupled GPCR, such as β-adrenoceptors by adrenergic agonists in human dermal microvascular endothelial cells [108], or prostanoid receptors by prostaglandin E2 (PGE2), and PGI2 in human pulmonary artery endothelial cells [109,110], and the subsequent formation of a cAMP pool within the subplasma membrane compartment by the action of transmembrane isoforms of AC, contribute significantly to the maintenance of the integrity of the endothelial barrier. Moreover, it should be noted that there is variability in the regulation of endothelial permeability in response to agonists, depending on the endothelial cell model used, as recently demonstrated in different phenotypes of human endothelial cells stimulated with histamine, platelet-activating factor (PAF), or thrombin [111].

The increase in cAMP derived from transmembrane ACs constitutes an intracellular signal that activates intercellular adhesin in the endothelium, preserving its integrity. AC6 has been related to the generation of cAMP for the maintenance of endothelial permeability. Using both pulmonary artery endothelial cells (PAECs) and pulmonary microvascular endothelial cells (PMVECs), Cioffi et al. [96] demonstrated that store-operated Ca^2+^ entry activated by thrombin inhibits AC6 activity, and the subsequent decrease in cAMP induces gap formation. In PMVECs, AC6 is located in caveolin-rich lipid rafts of the endothelial plasma membrane, and the cAMP formed in this environment causes the stabilization of actin, mediated by the phosphorylation of the protein filamin A, thus causing the reinforcement of the barrier function [112].

On the other hand, sAC activity is not modulated by G proteins, but stimulated by Ca^2+^, bicarbonate, and ATP [113,114,115]. sAC was first described in cytosolic extracts from frozen rat testis [116], and is also expressed in endothelium, where it can participate in various functions. For example, it plays a key role in ischemic- or acidic stress-induced apoptosis of coronary endothelial cells [117], regulates endothelial stiffness and actin fiber composition [118], and controls the calcium response dependent on the endoplasmic reticulum in human vascular endothelial cells, so it could constitute a pharmacological target for the treatment of cardiovascular diseases, such as heart failure or arterial hypertension [119].

Regarding endothelial permeability, many bacteria may generate soluble forms of AC that can act as effectors of virulence or toxins, allowing them to invade the immune system of the host [105]. These toxins include, for example, ExoY from Pseudomonas aeruginosa [101,120], which causes inter-endothelial cell gaps to form. Thus, pathogenic bacteria can enhance endothelial permeability of eukaryotic cells through cAMP synthesis [97,121].

**Figure 2:**
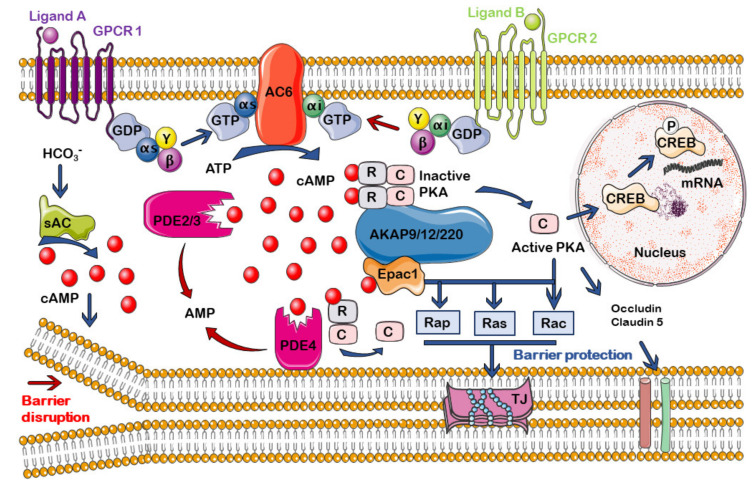
Suggested model of compartmentalization of the cAMP signal in endothelial cells of the cerebral microvasculature and its participation in the integrity of the barrier function. AC: adenylyl cyclase; AKAP: A-kinase anchor proteins; GPCR: G-protein coupled receptor; PDE: phosphodiesterase; PKA: protein kinase A; sAC: soluble adenylyl cyclase; TJ: Tight junction. For more details, see text.

The activation of ACs is involved in the different activity of cAMP on endothelial membrane function, depending on whether cAMP production takes place at the membrane or cytoplasmic level. While endogenous synthesis of cAMP near the membrane from transmembrane ACs protects endothelial cell barrier integrity and acts as an intracellular signal that favors adhesion [112], cytosolic cAMP pools generated by sAC give rise to a disruptive effect and increase permeability [93]. Although there are no studies in this regard, it can be hypothesized that a similar model may regulate endothelial permeability at the BBB (Figure 2). Thus, it could be assumed that selective activation of AC6 and/or selective inhibition of sAC may provide pharmacological protection for BBB integrity.

#### 6.1.2. cAMP Phosphodiesterases (PDEs)

PDEs are enzymes that hydrolyze cAMP and cyclic guanosine monophosphate (cGMP) by breaking down the phosphodiester bond. It is a superfamily of more than 100 proteins grouped into 11 families. Their great variety and intracellular distribution make them participate in the fine adjustment of the compartmentalized regulation of cGMP and cAMP. Regarding its substrate, PDE4, PDE7, and PDE8 are specific for cAMP. PDE1, PDE2, PDE3, PDE10, and PDE11 families hydrolyze both cGMP and cAMP. In the PDE1 family, only the PDE1C subtype degrades both cAMP and cGMP with similar Km values, while PDE1A and PDE1B are specific for cGMP. The other three families, PDE5, PDE6, and PDE9 only degrade cGMP [58,122,123].

PDEs play a fundamental role in the formation and maintenance of cAMP microdomains in various cell models [73]. Moreover, for the creation of cAMP nanocompartments in cells, the diffusion of cAMP needs to be restricted in the proximity of the PDEs, as demonstrated by the combined use of cAMP measurements using FRET, and a mathematical model of analysis based on PDE activity and cAMP diffusion [124].

Among the PDEs that hydrolyze cAMP, Ca^2+^/calmodulin-regulated PDE1, cGMP-stimulate PDE2, cGMP-inhibited PDE3, cAMP-specific PDE4, and cAMP-specific PDE7A are shown to express in endothelial cells, although, depending on the origin of the cells, significant differences have been found in their expression [123,125]. As far as the different PDEs playing a decisive role in the regulation of the amplitude, duration, and localization of cAMP in endothelial cells, they contribute to the regulation of barrier function, which is why abnormalities in their expressions or functioning can lead to pathological states [125] (Figure 2).

The role of PDE4 in regulating the permeability of the endothelial barrier and the BBB has been focused on [126,127]. In lung microvascular endothelium, the splice variant PDE4D4 is anchored to spectrin, a cytoskeletal protein located on the inner side of the plasma membrane. This PDE4D4 is responsible for orienting cAMP towards membrane domains where it activates barrier-enhancing effectors and prevents cAMP from accessing a cytosolic domain that is rich in microtubules, and where the phosphorylation of PKA induces cell gap formation [128]. It was also reported that vascular endothelial cadherins, which regulate vascular endothelial cell permeability, form complexes with Epac-1 by means of a selective interaction based on PDE4D. This process seems critical for the regulation of permeability in human aortic (HAEC) and microvascular cardiac (HMVEC-C) endothelial cells [129]. Furthermore, using rolipram, a selective PDE4 inhibitor, in a murine model of polymicrobial sepsis, Flemming et al. [130] found that PDE4 is involved in the alteration of the permeability of the endothelial barrier caused by inflammation during sepsis; thus, suggesting a potential clinical use for inhibitors of this enzyme.

Regarding BBB, the abilities of PDE4 inhibitors BBB022 and rolipram in reducing cerebrovascular endothelial permeability have also been demonstrated in the spinal cords of mice with experimental autoimmune encephalomyelitis, being able to prevent the entry of inflammatory cells and factors, and reduce tissue edema [131]. Moreover, by performing stroke experiments in mice, it was possible to demonstrate that rolipram maintains the expression of TJ proteins, such as occludin and claudin-5, avoiding ischemia-induced BBB disruption [132].

PDE2 also participates in the regulation of pulmonary endothelial permeability [127]. The increase in permeability induced by the pneumococcal exotoxin pneumolysin in isolated perfused mouse lungs and in human endothelial cell monolayers was reversed by selective inhibition of PDE2 with 9-(6-phenyl-2-oxohex-3-yl)-2-(3,4-dimethoxybenzyl)-purin-6one (PDP) or hydroxy-PDP [133].

Interestingly, the selective inhibition of PDE3 activity with cilostazol stabilized the barrier integrity in BBB-related endothelial cells (primary rat brain capillary endothelial cells and the human brain endothelial cell line hCMEC/D3) [134]. By means of TEER experiments, these authors reported that cilostazol protects BBB from damage induced by oxygen glucose deprivation (OGD) and reoxygenation and increases electrical resistance by improving the tightness of TJs. This barrier-enhancing effect was mediated by PKA, since it was reduced after its inhibition with H-89. As PDE3B is mainly expressed in these cells, the authors suggested an interest in the development of new inhibitors of this isoform, as neuroprotectors, avoiding BBB pathological permeability.

Consequently, PDE activity inhibition may serve to prevent the disruption of BBB, with PDE4 and PDE3 being prime candidates for this action. Although PDE2 inhibition cannot be ruled out, there are no studies in this regard at the level of the cerebral microvasculature.

### 6.2. A-Kinase Anchoring Proteins (AKAPs)

AKAPs are a family of proteins with great structural variety and with a wide capacity to scaffold combinations of signaling molecules. All AKAPs bind to the regulatory R subunits of PKA, assembling this protein in multiprotein signaling complexes and participating prominently in the compartmentalization of the cAMP signal. In fact, AKAP proteins are implicated in the spatial regulation of cAMP concentrations by interacting with β-adrenergic receptors, ACs, and PDEs [135]. In addition to PKA, AKAPs interact with many other cellular binding partners, including Epac proteins, thus contributing to the compartmentalization of cAMP-activated effectors [135,136].

AKAPs, due to their broad ability for spatial regulation, are involved in the regulation of a significant number of physiological functions (and, therefore, of potential pathological alterations). For this reason, they are postulated as possible drug targets, to develop precision pharmacology, which allows for more efficient drug targeting [38,136,137].

In the cardiovascular system, various AKAPs participate in regulatory functions at the cardiac and vasculature levels. They control, among other functions, cardiac stress response, cardiac repolarization, vascular integrity, peripheral arteries vasoconstriction, and endothelial barrier function [138,139].

Among the AKAPs involved in the regulation of endothelial permeability, the long isoform of AKAP9 is described as associating with microtubules of human endothelial cells, forming a complex with Epac1. Thus, AKAP9 is necessary for integrin-mediated adhesion in HUVEC cells, participating in the enhancement of cAMP-induced barrier function through the activation of Epac1 [140].

AKAP12, also known as gravin or src-suppressed *C*-kinase substrate (SSeCKS), was reported to participate in BBB differentiation by modulating both brain angiogenesis and TJ formation in human brain microvascular endothelial cells (HBMEC) [138,141]. This anchoring protein also induces blood–retinal barrier formation (a selective eye barrier composed of microvascular endothelial cells in a basement membrane, surrounded by astrocytes) in the developing human eye, by facilitating the formation of TJs [142]. Furthermore, the pro-barrierogenic functions of this protein were demonstrated in different cell models [138,143].

Another isoform, AKAP220, participates, together with AKAP12, in the regulation of the endothelial barrier. They contribute to the formation of a protein complex that links the cAMP signaling pathway with adherent junctions and is required for cAMP-mediated barrier stabilization in human dermal microvascular endothelial cells [143].

Taking the foregoing into account—AKAP9, AKAP12, and AKAP20 constitute potential pharmacological targets that could allow obtaining drugs designed to selectively protect the permeability of BBB, avoiding the progression of neurodegenerative diseases, such as AD, although more specific studies are needed.

### 6.3. cAMP Effectors: PKA and Epac

#### 6.3.1. PKA

PKA is a serine/threonine kinase whose activation by cAMP results in the phosphorylation of a vast number of substrates [136]. It has been widely studied from a structural and functional point of view; moreover, its relationship with the origin and evolution of various diseases, such as myocardial infarction [144], cancer [145], and obesity [146], among many others, has been studied. Furthermore, PKA participates in gene transcription through phosphorylation of the cAMP-response element binding protein (CREB) at the level of the cell nucleus [73]. In fact, it could be argued that the cAMP/PKA pathway is one of the most widely studied intracellular signaling pathways. In this sense, there are numerous studies concerning the participation of PKA in the spatial and temporal localization of cAMP signaling [73,94].

PKA consists of two regulatory subunits (RI and RII), each having α and β isoforms that maintain, and two catalytic (C) subunits in an inactive state (until their activation by cAMP). Within cells, PKA is commonly distributed in microdomains due to its association with the anchor protein AKAP. This association serves as the basis for the integration of multiple intracellular signalosomes, but also the spread of convergent signaling pathways. It should be noted that PKA is not necessarily bound to AKAP, but it can be soluble [94,147,148].

In relation to vascular endothelium, PKA has various functions. It acts as a mediator of cAMP synthesized by sAC to increase the proapoptotic activity of the Bax protein [117]. Regarding the endothelial barrier, in general, PKA is involved in the phosphorylation and expression of proteins implicated in the regulation of TJs in endothelial cells, such as occludin and claudin-5 [149] (Figure 2).

The spatial and temporal regulation of PKA-mediated signaling and its participation in the maintenance of endothelial barrier function is more dependent on the formation of multivalent complexes with AKAP. The compartmentalized activation of Rac1, an important signaling molecule for barrier stabilization by PKA, takes place in the vicinity of the anterior junctions and the cortical actin cytoskeleton due to its anchoring to AKAP220 [143].

It was reported that, in endothelial cells, a localized pool of cAMP is generated by the activation of transmembrane ACs and its diffusion is restricted by PDE4D4. The inhibition of PDE4D allow cAMP to access cytoskeletal targets and activate PKA that phosphorylates tau-serine 214, leading to a reorganization of microtubules and the induction of cell gaps [150].

#### 6.3.2. Exchange Proteins Directly Activated by cAMP (Epac)

The discovery of Epac, together with the appearance of drugs that selectively activate or inhibit it, has demonstrated its participation in various cellular functions previously attributed to the activation of PKA [151,152]. It has opened new horizons of research in the field of cAMP and cAMP-activated signaling pathways and is currently considered an emerging pharmacological target in various pathologies [153].

It is now accepted that Epac participates in multiple cellular functions mediated by cAMP, such as cell adhesion, cell–cell junctions, secretion/exocytosis, cell differentiation and proliferation, apoptosis, gene expression, cardiac hypertrophy, and phagocytosis [154]. In several processes, Epac can act in combination with PKA, exerting synergistic or antagonistic effects, which reveal an interconnectivity between these two cAMP-activated pathways [154,155,156].

Two isoforms of Epac have been described, Epac1 and Epac 2. They act as guanine exchange factors for Ras-like GTPases Rap1 and Rap2 [157,158]. Most studies have identified Epac1 as the principal isoform in vascular endothelial cells (see, i.e., [155,159]), although the existence of Epac2 has also been detected in some endothelial cell models and it was shown to be inducible in human microvascular endothelial cells [160].

There is strong evidence that Epac may play a more decisive role than PKA in regulating endothelial barrier function. A study by Rampersad et al. [129] identified the isoform Epac1 as the dominant cAMP effector operating permeability in both arterial and microvessel-derived human vascular endothelial cells. The expression of Epac1 is significantly reduced by hypoxia, resulting in endothelial hyperpermeability and NO/ROS imbalance, and the selective activation of Epac-1 with 8-pCPT-2′-*O*-Me-cAMP restores the endothelial barrier and stimulates endothelial nitric oxide synthase, increasing NO production [161]. This last effect can also contribute to the stabilization of the endothelial barrier and takes place through the phosphorylation of Ser 1177 activating the phosphoinositide 3-kinase/Akt pathway, an effect in which PKA would also participate synergistically [155]. By using an Epac1^−/−^ knockout mouse model, Kopperud et al. [162] demonstrated that Epac1 participates in this effect of cAMP, since it exerts a tonic inhibition of in vivo basal microvascular permeability. Moreover, it has been described that Epac (but not PKA) activation may reduce microvascular permeability and inflammation in LPS-induced lung injury [163].

More recently, a novel signaling pathway that reduces endothelial permeability in vitro and in vivo was described. In this circuit, the junctional adhesion molecule-A (JAM-A), which has a key role in the maintenance of endothelial barrier function, induces claudin-5 expression, a key component of the TJ strands, through a cAMP-activated Epac-dependent mechanism [164]. These data describe a regulatory pathway involving Epac, which allows regulation of vascular permeability through TJs.

A report by Ramos and Antonetti [165] suggested that activation of Epac by cAMP can restore barrier properties in the BBB after a breakdown. However, despite the already demonstrated role of cAMP levels in regulating the permeability of peripheral microvessels and BBB, the relative role of Epac proteins in this function is rarely known. On the contrary, it was also suggested that the cAMP-mediated increase in the gap junction coupling does not depend on the activation of PKA or Epac [69].

Epac proteins act as guanine-nucleotide-exchange factors (GEF) for the Ras-related proteins Rap1 and Rap2 [153]. The activation of Rap1 is directly involved in the maintenance of endothelial permeability and the regulation of two members of the Rho family of GTPases, Rac and RhoA, as well as that of the cytoskeleton protein ezrin [166].

In recent years, the role of the activation of GTPases by Epac in the control of endothelial permeability has been extensively studied (see, i.e., [160,167,168,169]), although the review of these mechanisms exceeds the purpose of the present work. Furthermore, Epac/Rap signaling and the role of small GTPases in the regulation of BBB has recently been reviewed [165].

Therefore, cAMP/Epac signaling pathway appears as a promising pathway to regulate BBB permeability, due to its great implication in maintaining low endothelial permeability. However, more specific studies are needed at the level of the brain microvasculature to confirm this possibility.

## 7. cAMP Compartmentalization in the BBB and Aging

It is generally accepted that BBB dysfunction occurs during aging and there is increasing evidence of its close relationship with cognitive impairment [170]. An abnormally high permeability of the BBB may constitute an early marker for predicting cognitive decline in AD progression. However, despite the prominent role that it plays in regulating endothelial permeability, knowledge about cAMP signaling pathways and compartmentalization at the BBB level is scarce, as can be seen in the previous sections.

Furthermore, there are almost no studies on cAMP signaling alterations during aging or neurodegenerative diseases. Consequently, at present, there are no studies on drugs that, acting on the different proteins involved in the compartmentalization of cAMP at the BBB, can provide a benefit in the treatment of AD. In any case, there are some studies that may serve as the basis for future studies on the influence of aging, on the regulation of cAMP signalosomes in the BBB.

It is important to note that most studies in this regard refer to the influence of age on the cAMP compartmentalization in neurons, not in the BBB, which is out of the scope of this review. With aging, there is a significant decrease in AC activity in both human and animal brains, leading to lower levels of cAMP, which may favor the development of neurodegenerative diseases. Thus, during aging, the local distribution of the cAMP signal in neurons is altered, a situation that can participate in the development of various diseases, including neurodegenerative processes, such as AD [57]. However, there are no specific studies on cerebral microvasculature, demonstrating a similar decrease in AC activity or an alteration in its intracellular distribution that could lead to a progressive increase in the permeability of the BBB with aging.

There are few studies on the alteration of the expression of PDE in the brain during aging, which showed contradictory results [57,171]. Moreover, cilostazol treatment has been shown to exert beneficial long-term effects, reducing age-related cognitive decline in senescence accelerated mouse (SAMP8), a mouse model of cognitive aging. This effect is exerted by a mechanism related to the increase of cAMP in the brain and the protection of the integrity of the BBB [172]. The role of PDEs in endothelial function in ischemic stroke, including regulation of permeability, has recently been reviewed [135].

Similarly, the possible pathological alterations in the function of AKAPs during aging and their implication in cognitive deterioration have hardly been studied and there are no studies of this type at the endothelial barrier level [13].

Finally, it was reported that cAMP-mediated relaxation in mice basilar arteries is mainly related to the activation of Epac versus PKA, and is diminished with endothelial and smooth muscle aging [173]. However, as with other elements of the cAMP signaling pathway, most of the studies on the possible influence of Epac alterations in aged-related neurodegenerative diseases concerns its presence in the brain and there are no studies of its possible alteration at the BBB level [171].

## 8. Conclusions and Perspectives

In the search for new, successful, therapeutic options to treat AD, the vascular hypothesis paves the way for potential strategies related to the improvement of blood flow at the cerebral microvascular and protection of the BBB, as highlighted in this review.

Preserving the permeability of this barrier—or even achieving a hypothetical reversal of existing pathological disruptions—could contribute toward slowing, or even stopping, the progression of neurodegenerative diseases, including primary degenerative dementias, such as AD.

The important role that cAMP plays in maintaining the endothelial barrier function is widely known. However, the compartmentalization of cAMP signaling has certainly been studied more in other barriers, such as pulmonary or retinal, or endothelial functioning, in general, using various animal or human cell models. As each barrier has its own peculiarities, both at the functional and molecular level, it seems necessary to gather more knowledge on the cAMP signaling pathway at the BBB, as well as on the spatial and temporal distribution of this signal at the endothelium that integrates this barrier.

The distribution of the different pools of cAMP has also been studied at the brain level. However, a therapeutic proposal based on the vascular hypothesis and the protection of the BBB for the treatment of AD involves the delivery of drugs to a target in the endothelium of the cerebral microvasculature. Therefore, it would not be necessary to use molecules that must cross the BBB towards the SNC.

The ubiquity of cAMP means that less selective drugs can exert actions in different areas of the body, which increases the risk of adverse effects. Therefore, the use of more selective drugs, targeting the various protein isoforms directly involved in regulating BBB permeability, represents a potentially more effective and safe strategy. In this sense, a new therapeutic approach to modify the course of the disease by reducing BBB permeability could include the enhancement of certain cAMP intracellular pools through pharmacological actions on different subtypes of ACs and PDEs, the selective activation or inhibition of PKA or Epac subtypes, or a strategy to induce the formation of certain molecular clusters by acting on anchor proteins, such as AKAP.

In conclusion, developing a new strategy to treat AD, based on the ability of cAMP in protecting BBB integrity against disrupting pathogenic agents, makes it essential to gather more knowledge on i) the compartmentalization of cAMP-dependent processes that regulate BBB permeability; and ii) the development of molecules that can selectively modulate the agents involved in this compartmentalization and/or that can be directed in a more selective way towards the BBB.
